# Supplementary material for: A Likelihood Approach for Real-Time Calibration of Stochastic Compartmental Epidemic Models
Source: PLoS Comput Biol. 2017 Jan 17;13(1):e1005257. doi: 10.1371/journal.pcbi.1005257 (PMC5240920; doi:10.1371/journal.pcbi.1005257)
Supplement: S1 File — (TAR.GZ) [file pcbi.1005257.s014.tar.gz › HSPH_Online-SI-Revision/EnKF07/n10-extreme/n10-extreme_PF2_EnKF-summary2.pdf]

peak -8	3w spec	3w cum	5w spec	5w cum	end	3w spec LOG	3w cum LOG	5w spec LOG	5w cum LOG	end LOG
mean	430.8	1858.4	83.6	346.8	56.1	0.9	1.	1.5	0.6	0.6
median	251.3	1283.6	85.	287.6	56.4	0.8	1.	1.3	0.6	0.5
quantile .1	86.8	82.1	65.2	73.6	32.1	0.5	0.4	0.7	0.4	0.2
quantile .9	809.9	3542.1	98.5	747.1	88.7	1.2	1.4	2.3	0.4	1.3
39	8	21	21	38						

peak -4	3w spec	3w cum	5w spec	5w cum	end	3w spec LOG	3w cum LOG	5w spec LOG	5w cum LOG	end LOG
mean	88.5	91.1	93.9	80.5	79.	1.6	0.7	2.4	0.9	1.1
median	90.9	92.2	94.7	84.3	85.3	1.6	0.8	2.5	1.	1.2
quantile .1	69.1	71.6	88.5	60.3	57.	0.7	0.3	1.4	0.4	0.5
quantile .9	98.4	108.	97.6	93.5	89.4	2.2	1.1	2.9	0.4	1.4
3	36	0	26	12						

peak	3w spec	3w cum	5w spec	5w cum	end	3w spec LOG	3w cum LOG	5w spec LOG	5w cum LOG	end LOG
mean	93.8	93.1	96.4	90.6	88.6	1.7	0.9	2.1	0.9	1.
median	94.7	92.8	97.3	90.5	88.9	1.6	0.9	2.3	0.9	1.
quantile .1	87.6	76.	90.4	76.2	75.6	1.	0.5	1.4	0.6	0.7
quantile .9	101.3	106.5	100.7	100.	95.8	2.4	1.1	2.7	0.6	1.3
6	25	1	20	19						

peak +4	3w spec	3w cum	5w spec	5w cum	end	3w spec LOG	3w cum LOG	5w spec LOG	5w cum LOG	end LOG
mean	102.6	107.9	113.3	105.2	107.3	1.3	0.6	1.5	0.6	0.6
median	99.7	96.4	101.5	95.8	99.4	1.3	0.5	1.5	0.6	0.6
quantile .1	77.3	71.7	80.3	71.3	74.9	0.6	0.3	0.8	0.3	0.4
quantile .9	119.2	158.9	139.8	144.	150.4	1.9	0.8	2.	0.3	0.8
22	39	18	37	38						

peak +8	3w spec	3w cum	5w spec	5w cum	end	3w spec LOG	3w cum LOG	5w spec LOG	5w cum LOG	end LOG
mean	409.2	676.8	801.8	630.1	678.5	1.	0.5	1.2	0.5	0.5
median	201.7	440.6	266.1	363.8	348.	0.9	0.5	1.3	0.5	0.5
quantile .1	75.9	125.1	85.5	135.6	137.8	0.4	0.3	0.7	0.3	0.3
quantile .9	1038.6	1776.9	1627.5	1570.6	1900.6	1.5	0.8	1.6	0.3	0.8
46	38	42	40	39	{}	{}	{}	{}	{}	{}
